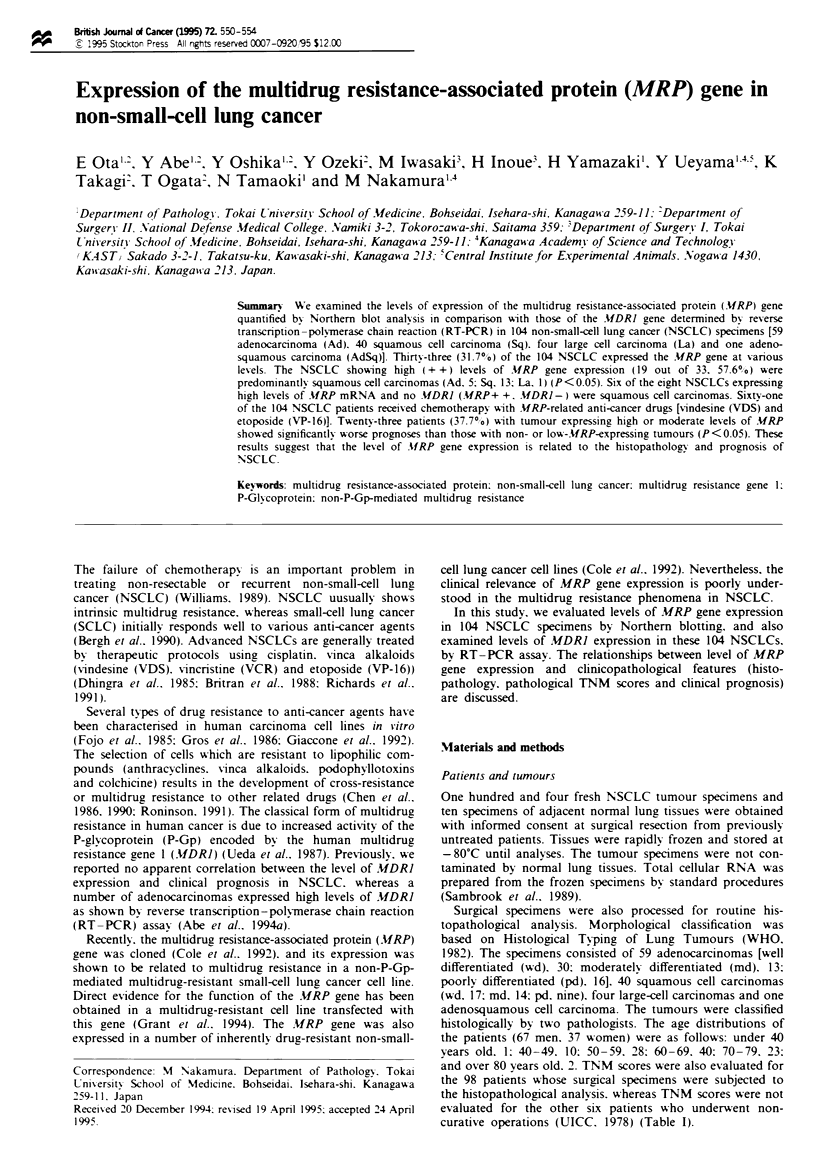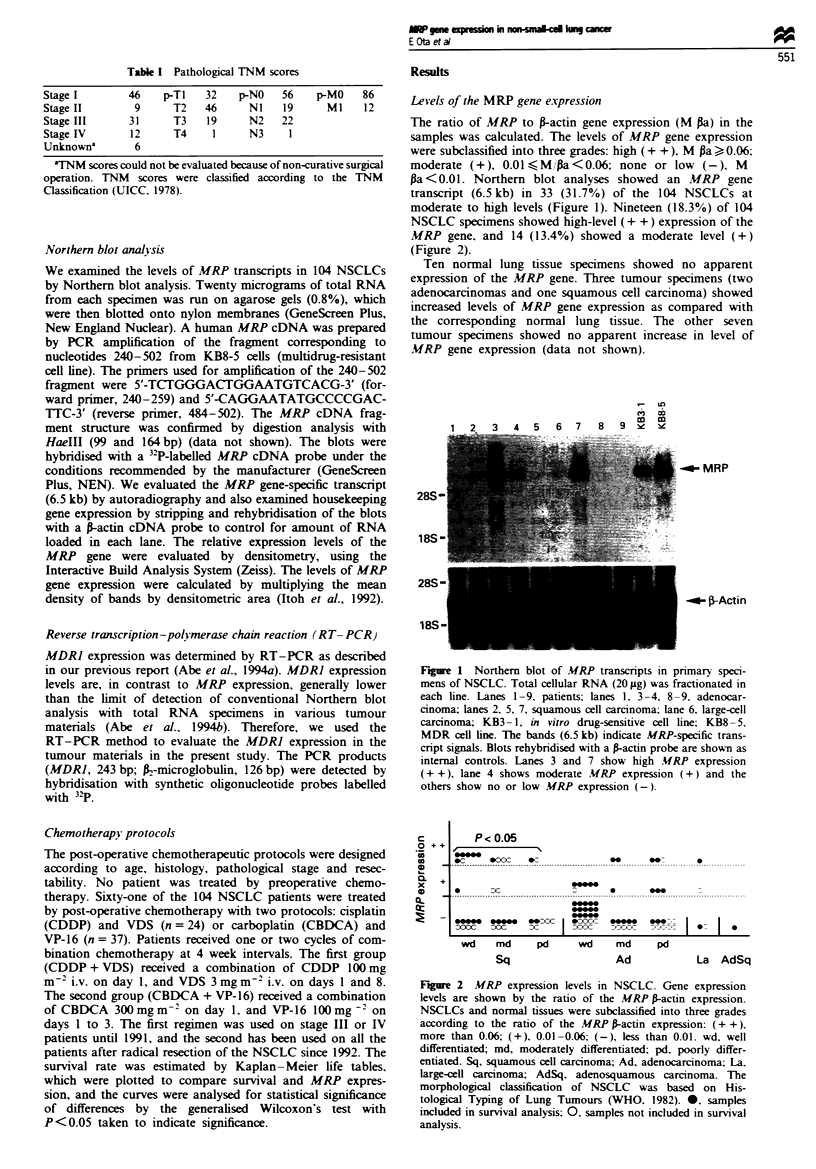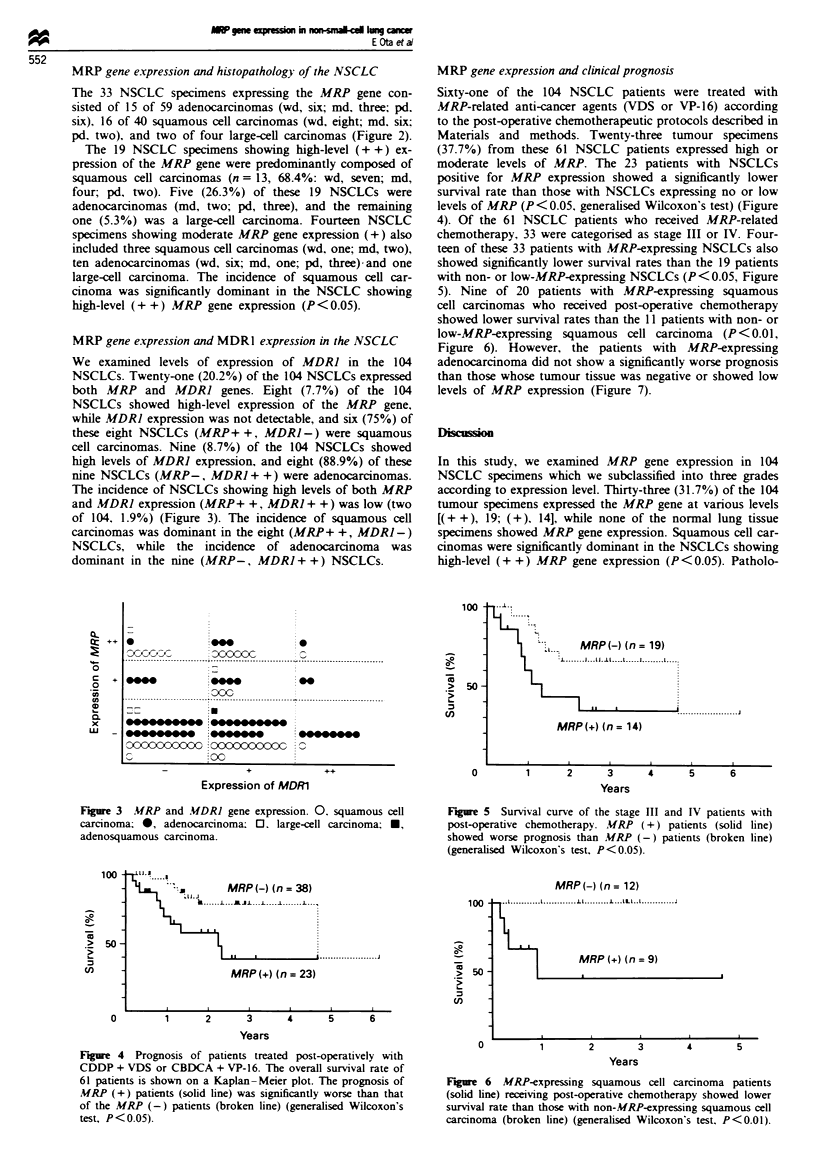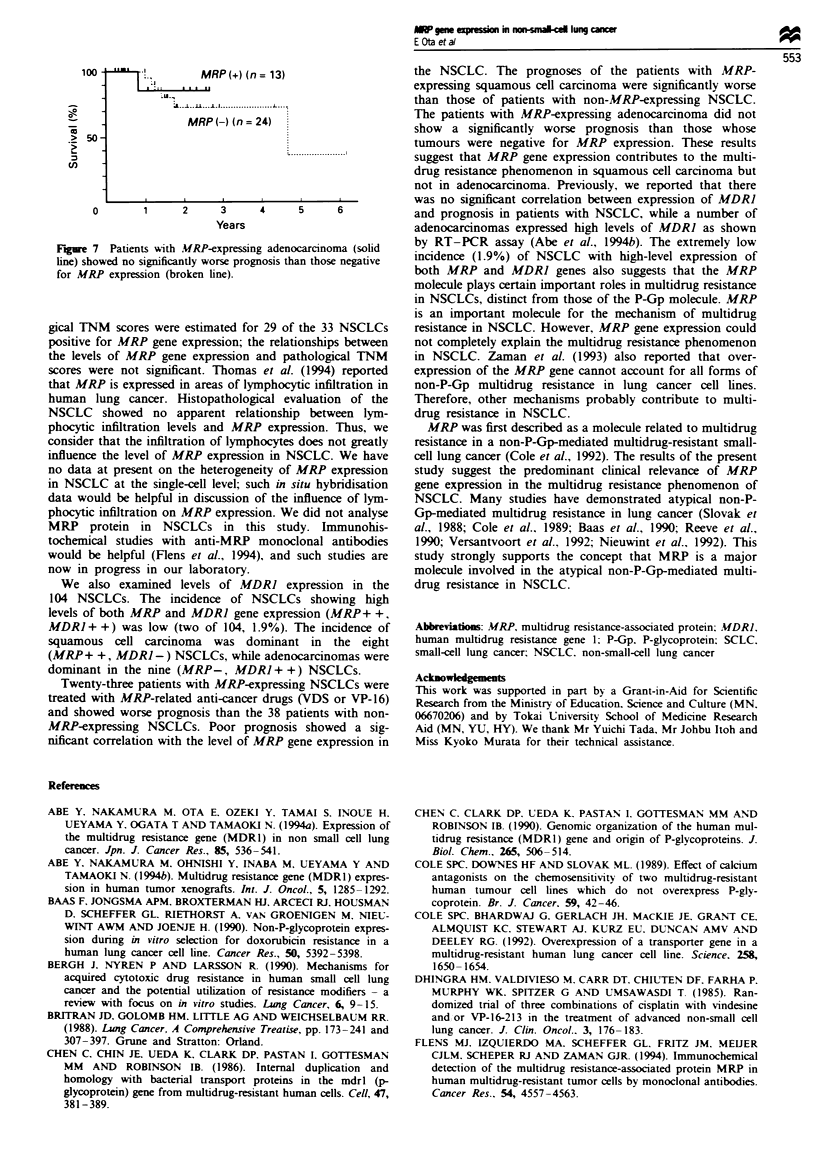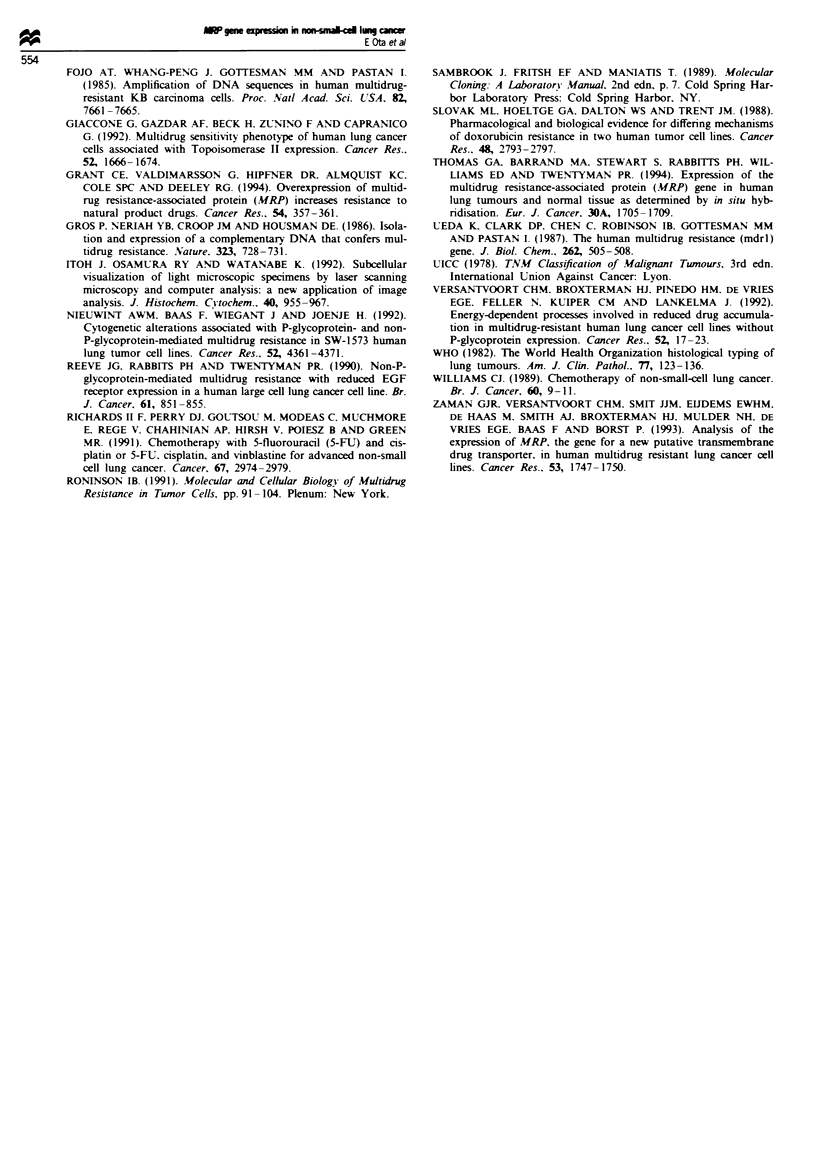# Expression of the multidrug resistance-associated protein (MRP) gene in non-small-cell lung cancer.

**DOI:** 10.1038/bjc.1995.372

**Published:** 1995-09

**Authors:** E. Ota, Y. Abe, Y. Oshika, Y. Ozeki, M. Iwasaki, H. Inoue, H. Yamazaki, Y. Ueyama, K. Takagi, T. Ogata

**Affiliations:** Department of Pathology, Tokai University School of Medicine, Kanagawa, Japan.

## Abstract

**Images:**


British Journal of Cancer (1995) 72, 550-554

© 1995 Stockton Press All rights reserved 0007-0920/95 $12.00

### Expression of the multidrug resistance-associated protein (*MRP*) gene in non-small-cell lung cancer

E Ota[1,2], Y Abe[1,2], Y Oshika[1,2], Y Ozeki[2], M Iwasaki[3], H Inoue[3], H Yamazaki[1], Y Ueyama[1,4,5], K Takagi[2], T Ogata[2], N Tamaoki[1] and M Nakamura[1,4]

[1]*Department of Pathology, Tokai University School of Medicine, Bohseidai, Isehara-shi, Kanagawa 259-11;* [2]*Department of Surgery II, National Defense Medical College, Namiki 3-2, Tokorozawa-shi, Saitama 359;* [3]*Department of Surgery I, Tokai University School of Medicine, Bohseidai, Isehara-shi, Kanagawa 259-11;* [4]*Kanagawa Academy of Science and Technology (KAST) Sakado 3-2-1, Takatsu-ku, Kawasaki-shi, Kanagawa 213;* [5]*Central Institute for Experimental Animals, Nogawa 1430, Kawasaki-shi, Kanagawa 213, Japan.*

**Summary** We examined the levels of expression of the multidrug resistance-associated protein (*MRP*) gene quantified by Northern blot analysis in comparison with those of the *MDR1* gene determined by reverse transcription-polymerase chain reaction (RT-PCR) in 104 non-small-cell lung cancer (NSCLC) specimens [59 adenocarcinoma (Ad), 40 squamous cell carcinoma (Sq), four large cell carcinoma (La) and one adenosquamous carcinoma (AdSq)]. Thirty-three (31.7%) of the 104 NSCLC expressed the *MRP* gene at various levels. The NSCLC showing high (++) levels of *MRP* gene expression (19 out of 33, 57.6%) were predominantly squamous cell carcinomas (Ad, 5; Sq, 13; La, 1) ($P<0.05$). Six of the eight NSCLCs expressing high levels of *MRP* mRNA and no *MDR1* (*MRP*++, *MDR1*−) were squamous cell carcinomas. Sixty-one of the 104 NSCLC patients received chemotherapy with *MRP*-related anti-cancer drugs [vindesine (VDS) and etoposide (VP-16)]. Twenty-three patients (37.7%) with tumour expressing high or moderate levels of *MRP* showed significantly worse prognoses than those with non- or low-*MRP*-expressing tumours ($P<0.05$). These results suggest that the level of *MRP* gene expression is related to the histopathology and prognosis of NSCLC.

**Keywords**: multidrug resistance-associated protein; non-small-cell lung cancer; multidrug resistance gene 1; P-Glycoprotein; non-P-Gp-mediated multidrug resistance

The failure of chemotherapy is an important problem in treating non-resectable or recurrent non-small-cell lung cancer (NSCLC) (Williams, 1989). NSCLC uusually shows intrinsic multidrug resistance, whereas small-cell lung cancer (SCLC) initially responds well to various anti-cancer agents (Bergh *et al.*, 1990). Advanced NSCLCs are generally treated by therapeutic protocols using cisplatin, vinca alkaloids (vindesine (VDS), vincristine (VCR) and etoposide (VP-16)) (Dhingra *et al.*, 1985; Britran *et al.*, 1988; Richards *et al.*, 1991).

Several types of drug resistance to anti-cancer agents have been characterised in human carcinoma cell lines *in vitro* (Fojo *et al.*, 1985; Gros *et al.*, 1986; Giaccone *et al.*, 1992). The selection of cells which are resistant to lipophilic compounds (anthracyclines, vinca alkaloids, podophyllotoxins and colchicine) results in the development of cross-resistance or multidrug resistance to other related drugs (Chen *et al.*, 1986, 1990; Roninson, 1991). The classical form of multidrug resistance in human cancer is due to increased activity of the P-glycoprotein (P-Gp) encoded by the human multidrug resistance gene 1 (*MDR1*) (Ueda *et al.*, 1987). Previously, we reported no apparent correlation between the level of *MDR1* expression and clinical prognosis in NSCLC, whereas a number of adenocarcinomas expressed high levels of *MDR1* as shown by reverse transcription-polymerase chain reaction (RT-PCR) assay (Abe *et al.*, 1994*a*).

Recently, the multidrug resistance-associated protein (*MRP*) gene was cloned (Cole *et al.*, 1992), and its expression was shown to be related to multidrug resistance in a non-P-Gp-mediated multidrug-resistant small-cell lung cancer cell line. Direct evidence for the function of the *MRP* gene has been obtained in a multidrug-resistant cell line transfected with this gene (Grant *et al.*, 1994). The *MRP* gene was also expressed in a number of inherently drug-resistant non-small-cell lung cancer cell lines (Cole *et al.*, 1992). Nevertheless, the clinical relevance of *MRP* gene expression is poorly understood in the multidrug resistance phenomena in NSCLC.

In this study, we evaluated levels of *MRP* gene expression in 104 NSCLC specimens by Northern blotting, and also examined levels of *MDR1* expression in these 104 NSCLCs, by RT-PCR assay. The relationships between level of *MRP* gene expression and clinicopathological features (histopathology, pathological TNM scores and clinical prognosis) are discussed.

### Materials and methods

### *Patients and tumours*

One hundred and four fresh NSCLC tumour specimens and ten specimens of adjacent normal lung tissues were obtained with informed consent at surgical resection from previously untreated patients. Tissues were rapidly frozen and stored at −80°C until analyses. The tumour specimens were not contaminated by normal lung tissues. Total cellular RNA was prepared from the frozen specimens by standard procedures (Sambrook *et al.*, 1989).

Surgical specimens were also processed for routine histopathological analysis. Morphological classification was based on Histological Typing of Lung Tumours (WHO, 1982). The specimens consisted of 59 adenocarcinomas [well differentiated (wd), 30; moderately differentiated (md), 13; poorly differentiated (pd), 16], 40 squamous cell carcinomas (wd, 17; md, 14; pd, nine), four large-cell carcinomas and one adenosquamous cell carcinoma. The tumours were classified histologically by two pathologists. The age distributions of the patients (67 men, 37 women) were as follows: under 40 years old, 1; 40-49, 10; 50-59, 28; 60-69, 40; 70-79, 23; and over 80 years old, 2. TNM scores were also evaluated for the 98 patients whose surgical specimens were subjected to the histopathological analysis, whereas TNM scores were not evaluated for the other six patients who underwent non-curative operations (UICC, 1978) (Table I).

Correspondence: M Nakamura, Department of Pathology, Tokai University School of Medicine, Bohseidai, Isehara-shi, Kanagawa 259-11, Japan
Received 20 December 1994; revised 19 April 1995; accepted 24 April 1995.

**Table I** Pathological TNM scores

| | | | | | | | |
|---|---|---|---|---|---|---|---|
| Stage I | 46 | p-T1 | 32 | p-N0 | 56 | p-M0 | 86 |
| Stage II | 9 | T2 | 46 | N1 | 19 | M1 | 12 |
| Stage III | 31 | T3 | 19 | N2 | 22 | | |
| Stage IV | 12 | T4 | 1 | N3 | 1 | | |
| Unknown[a] | 6 | | | | | | |

[a]TNM scores could not be evaluated because of non-curative surgical operation. TNM scores were classified according to the TNM Classification (UICC, 1978).

*Northern blot analysis*

We examined the levels of *MRP* transcripts in 104 NSCLCs by Northern blot analysis. Twenty micrograms of total RNA from each specimen was run on agarose gels (0.8%), which were then blotted onto nylon membranes (GeneScreen Plus, New England Nuclear). A human *MRP* cDNA was prepared by PCR amplification of the fragment corresponding to nucleotides 240–502 from KB8-5 cells (multidrug-resistant cell line). The primers used for amplification of the 240–502 fragment were 5′-TCTGGGACTGGAATGTCACG-3′ (forward primer, 240–259) and 5′-CAGGAATATGCCCCGACTTC-3′ (reverse primer, 484–502). The *MRP* cDNA fragment structure was confirmed by digestion analysis with *Hae*III (99 and 164 bp) (data not shown). The blots were hybridised with a $^{32}$P-labelled *MRP* cDNA probe under the conditions recommended by the manufacturer (GeneScreen Plus, NEN). We evaluated the *MRP* gene-specific transcript (6.5 kb) by autoradiography and also examined housekeeping gene expression by stripping and rehybridisation of the blots with a β-actin cDNA probe to control for amount of RNA loaded in each lane. The relative expression levels of the *MRP* gene were evaluated by densitometry, using the Interactive Build Analysis System (Zeiss). The levels of *MRP* gene expression were calculated by multiplying the mean density of bands by densitometric area (Itoh *et al*., 1992).

*Reverse transcription–polymerase chain reaction (RT–PCR)*

*MDR1* expression was determined by RT–PCR as described in our previous report (Abe *et al*., 1994*a*). *MDR1* expression levels are, in contrast to *MRP* expression, generally lower than the limit of detection of conventional Northern blot analysis with total RNA specimens in various tumour materials (Abe *et al*., 1994*b*). Therefore, we used the RT–PCR method to evaluate the *MDR1* expression in the tumour materials in the present study. The PCR products (*MDR1*, 243 bp; $\beta_2$-microglobulin, 126 bp) were detected by hybridisation with synthetic oligonucleotide probes labelled with $^{32}$P.

*Chemotherapy protocols*

The post-operative chemotherapeutic protocols were designed according to age, histology, pathological stage and resectability. No patient was treated by preoperative chemotherapy. Sixty-one of the 104 NSCLC patients were treated by post-operative chemotherapy with two protocols: cisplatin (CDDP) and VDS ($n = 24$) or carboplatin (CBDCA) and VP-16 ($n = 37$). Patients received one or two cycles of combination chemotherapy at 4 week intervals. The first group (CDDP + VDS) received a combination of CDDP 100 mg m$^{-2}$ i.v. on day 1, and VDS 3 mg m$^{-2}$ i.v. on days 1 and 8. The second group (CBDCA + VP-16) received a combination of CBDCA 300 mg m$^{-2}$ on day 1, and VP-16 100 mg $^{-2}$ on days 1 to 3. The first regimen was used on stage III or IV patients until 1991, and the second has been used on all the patients after radical resection of the NSCLC since 1992. The survival rate was estimated by Kaplan–Meier life tables, which were plotted to compare survival and *MRP* expression, and the curves were analysed for statistical significance of differences by the generalised Wilcoxon's test with $P < 0.05$ taken to indicate significance.

### Results

*Levels of the* MRP *gene expression*

The ratio of *MRP* to β-actin gene expression (M/βa) in the samples was calculated. The levels of *MRP* gene expression were subclassified into three grades: high (+ +), M/βa ≥ 0.06; moderate (+), 0.01 ≤ M/βa < 0.06; none or low (−), M/βa < 0.01. Northern blot analyses showed an *MRP* gene transcript (6.5 kb) in 33 (31.7%) of the 104 NSCLCs at moderate to high levels (Figure 1). Nineteen (18.3%) of 104 NSCLC specimens showed high-level (+ +) expression of the *MRP* gene, and 14 (13.4%) showed a moderate level (+) (Figure 2).

Ten normal lung tissue specimens showed no apparent expression of the *MRP* gene. Three tumour specimens (two adenocarcinomas and one squamous cell carcinoma) showed increased levels of *MRP* gene expression as compared with the corresponding normal lung tissue. The other seven tumour specimens showed no apparent increase in level of *MRP* gene expression (data not shown).

**Figure 1** Northern blot of *MRP* transcripts in primary specimens of NSCLC. Total cellular RNA (20 μg) was fractionated in each line. Lanes 1–9, patients; lanes 1, 3–4, 8–9, adenocarcinoma; lanes 2, 5, 7, squamous cell carcinoma; lane 6, large-cell carcinoma; KB3–1, *in vitro* drug-sensitive cell line; KB8–5, MDR cell line. The bands (6.5 kb) indicate *MRP*-specific transcript signals. Blots rehybridised with a β-actin probe are shown as internal controls. Lanes 3 and 7 show high *MRP* expression (+ +), lane 4 shows moderate *MRP* expression (+) and the others show no or low *MRP* expression (−).

**Figure 2** *MRP* expression levels in NSCLC. Gene expression levels are shown by the ratio of the *MRP*/β-actin expression. NSCLCs and normal tissues were subclassified into three grades according to the ratio of the *MRP*/β-actin expression: (+ +), more than 0.06; (+), 0.01–0.06; (−), less than 0.01. wd, well differentiated; md, moderately differentiated; pd, poorly differentiated. Sq, squamous cell carcinoma; Ad, adenocarcinoma; La, large-cell carcinoma; AdSq, adenosquamous carcinoma. The morphological classification of NSCLC was based on Histological Typing of Lung Tumours (WHO, 1982). ●, samples included in survival analysis; O, samples not included in survival analysis.

### MRP *gene expression and histopathology of the NSCLC*

The 33 NSCLC specimens expressing the *MRP* gene consisted of 15 of 59 adenocarcinomas (wd, six; md, three; pd, six), 16 of 40 squamous cell carcinomas (wd, eight; md, six; pd, two), and two of four large-cell carcinomas (Figure 2).

The 19 NSCLC specimens showing high-level (+ +) expression of the *MRP* gene were predominantly composed of squamous cell carcinomas ($n = 13$, 68.4%: wd, seven; md, four; pd, two). Five (26.3%) of these 19 NSCLCs were adenocarcinomas (md, two; pd, three), and the remaining one (5.3%) was a large-cell carcinoma. Fourteen NSCLC specimens showing moderate *MRP* gene expression (+) also included three squamous cell carcinomas (wd, one; md, two), ten adenocarcinomas (wd, six; md, one; pd, three) and one large-cell carcinoma. The incidence of squamous cell carcinoma was significantly dominant in the NSCLC showing high-level (+ +) *MRP* gene expression ($P < 0.05$).

### MRP *gene expression and* MDR1 *expression in the NSCLC*

We examined levels of expression of *MDR1* in the 104 NSCLCs. Twenty-one (20.2%) of the 104 NSCLCs expressed both *MRP* and *MDR1* genes. Eight (7.7%) of the 104 NSCLCs showed high-level expression of the *MRP* gene, while *MDR1* expression was not detectable, and six (75%) of these eight NSCLCs (*MRP*+ +, *MDR1*−) were squamous cell carcinomas. Nine (8.7%) of the 104 NSCLCs showed high levels of *MDR1* expression, and eight (88.9%) of these nine NSCLCs (*MRP*−, *MDR1*+ +) were adenocarcinomas. The incidence of NSCLCs showing high levels of both *MRP* and *MDR1* expression (*MRP*+ +, *MDR1*+ +) was low (two of 104, 1.9%) (Figure 3). The incidence of squamous cell carcinomas was dominant in the eight (*MRP*+ +, *MDR1*−) NSCLCs, while the incidence of adenocarcinoma was dominant in the nine (*MRP*−, *MDR1*+ +) NSCLCs.

### MRP *gene expression and clinical prognosis*

Sixty-one of the 104 NSCLC patients were treated with *MRP*-related anti-cancer agents (VDS or VP-16) according to the post-operative chemotherapeutic protocols described in Materials and methods. Twenty-three tumour specimens (37.7%) from these 61 NSCLC patients expressed high or moderate levels of *MRP*. The 23 patients with NSCLCs positive for *MRP* expression showed a significantly lower survival rate than those with NSCLCs expressing no or low levels of *MRP* ($P < 0.05$, generalised Wilcoxon's test) (Figure 4). Of the 61 NSCLC patients who received *MRP*-related chemotherapy, 33 were categorised as stage III or IV. Fourteen of these 33 patients with *MRP*-expressing NSCLCs also showed significantly lower survival rates than the 19 patients with non- or low-*MRP*-expressing NSCLCs ($P < 0.05$, Figure 5). Nine of 20 patients with *MRP*-expressing squamous cell carcinomas who received post-operative chemotherapy showed lower survival rates than the 11 patients with non- or low-*MRP*-expressing squamous cell carcinoma ($P < 0.01$, Figure 6). However, the patients with *MRP*-expressing adenocarcinoma did not show a significantly worse prognosis than those whose tumour tissue was negative or showed low levels of *MRP* expression (Figure 7).

### Discussion

In this study, we examined *MRP* gene expression in 104 NSCLC specimens which we subclassified into three grades according to expression level. Thirty-three (31.7%) of the 104 tumour specimens expressed the *MRP* gene at various levels [(+ +), 19; (+), 14], while none of the normal lung tissue specimens showed *MRP* gene expression. Squamous cell carcinomas were significantly dominant in the NSCLCs showing high-level (+ +) *MRP* gene expression ($P < 0.05$). Patholo-

**Figure 3** *MRP* and *MDR1* gene expression. ○, squamous cell carcinoma; ●, adenocarcinoma; □, large-cell carcinoma; ■, adenosquamous carcinoma.

**Figure 4** Prognosis of patients treated post-operatively with CDDP + VDS or CBDCA + VP-16. The overall survival rate of 61 patients is shown on a Kaplan–Meier plot. The prognosis of *MRP* (+) patients (solid line) was significantly worse than that of the *MRP* (−) patients (broken line) (generalised Wilcoxon's test, $P < 0.05$).

**Figure 5** Survival curve of the stage III and IV patients with post-operative chemotherapy. *MRP* (+) patients (solid line) showed worse prognosis than *MRP* (−) patients (broken line) (generalised Wilcoxon's test, $P < 0.05$).

**Figure 6** *MRP*-expressing squamous cell carcinoma patients (solid line) receiving post-operative chemotherapy showed lower survival rate than those with non-*MRP*-expressing squamous cell carcinoma (broken line) (generalised Wilcoxon's test, $P < 0.01$).

**Figure 7** Patients with *MRP*-expressing adenocarcinoma (solid line) showed no significantly worse prognosis than those negative for *MRP* expression (broken line).

gical TNM scores were estimated for 29 of the 33 NSCLCs positive for *MRP* gene expression; the relationships between the levels of *MRP* gene expression and pathological TNM scores were not significant. Thomas *et al.* (1994) reported that *MRP* is expressed in areas of lymphocytic infiltration in human lung cancer. Histopathological evaluation of the NSCLC showed no apparent relationship between lymphocytic infiltration levels and *MRP* expression. Thus, we consider that the infiltration of lymphocytes does not greatly influence the level of *MRP* expression in NSCLC. We have no data at present on the heterogeneity of *MRP* expression in NSCLC at the single-cell level; such *in situ* hybridisation data would be helpful in discussion of the influence of lymphocytic infiltration on *MRP* expression. We did not analyse MRP protein in NSCLCs in this study. Immunohistochemical studies with anti-MRP monoclonal antibodies would be helpful (Flens *et al.*, 1994), and such studies are now in progress in our laboratory.

We also examined levels of *MDR1* expression in the 104 NSCLCs. The incidence of NSCLCs showing high levels of both *MRP* and *MDR1* gene expression (*MRP*+ +, *MDR1*+ +) was low (two of 104, 1.9%). The incidence of squamous cell carcinoma was dominant in the eight (*MRP*+ +, *MDR1*–) NSCLCs, while adenocarcinomas were dominant in the nine (*MRP*–, *MDR1*+ +) NSCLCs.

Twenty-three patients with *MRP*-expressing NSCLCs were treated with *MRP*-related anti-cancer drugs (VDS or VP-16) and showed worse prognosis than the 38 patients with non-*MRP*-expressing NSCLCs. Poor prognosis showed a significant correlation with the level of *MRP* gene expression in the NSCLC. The prognoses of the patients with *MRP*-expressing squamous cell carcinoma were significantly worse than those of patients with non-*MRP*-expressing NSCLC. The patients with *MRP*-expressing adenocarcinoma did not show a significantly worse prognosis than those whose tumours were negative for *MRP* expression. These results suggest that *MRP* gene expression contributes to the multidrug resistance phenomenon in squamous cell carcinoma but not in adenocarcinoma. Previously, we reported that there was no significant correlation between expression of *MDR1* and prognosis in patients with NSCLC, while a number of adenocarcinomas expressed high levels of *MDR1* as shown by RT–PCR assay (Abe *et al.*, 1994*b*). The extremely low incidence (1.9%) of NSCLC with high-level expression of both *MRP* and *MDR1* genes also suggests that the *MRP* molecule plays certain important roles in multidrug resistance in NSCLCs, distinct from those of the P-Gp molecule. *MRP* is an important molecule for the mechanism of multidrug resistance in NSCLC. However, *MRP* gene expression could not completely explain the multidrug resistance phenomenon in NSCLC. Zaman *et al.* (1993) also reported that overexpression of the *MRP* gene cannot account for all forms of non-P-Gp multidrug resistance in lung cancer cell lines. Therefore, other mechanisms probably contribute to multidrug resistance in NSCLC.

*MRP* was first described as a molecule related to multidrug resistance in a non-P-Gp-mediated multidrug-resistant small-cell lung cancer (Cole *et al.*, 1992). The results of the present study suggest the predominant clinical relevance of *MRP* gene expression in the multidrug resistance phenomenon of NSCLC. Many studies have demonstrated atypical non-P-Gp-mediated multidrug resistance in lung cancer (Slovak *et al.*, 1988; Cole *et al.*, 1989; Baas *et al.*, 1990; Reeve *et al.*, 1990; Versantvoort *et al.*, 1992; Nieuwint *et al.*, 1992). This study strongly supports the concept that MRP is a major molecule involved in the atypical non-P-Gp-mediated multidrug resistance in NSCLC.

**Abbreviations**: *MRP*, multidrug resistance-associated protein; *MDR1*, human multidrug resistance gene 1; P-Gp, P-glycoprotein; SCLC, small-cell lung cancer; NSCLC, non-small-cell lung cancer

**Acknowledgements**
This work was supported in part by a Grant-in-Aid for Scientific Research from the Ministry of Education, Science and Culture (MN, 06670206) and by Tokai University School of Medicine Research Aid (MN, YU, HY). We thank Mr Yuichi Tada, Mr Johbu Itoh and Miss Kyoko Murata for their technical assistance.

### References

ABE Y, NAKAMURA M, OTA E, OZEKI Y, TAMAI S, INOUE H, UEYAMA Y, OGATA T AND TAMAOKI N. (1994*a*). Expression of the multidrug resistance gene (MDR1) in non small cell lung cancer. *Jpn. J. Cancer Res.*, **85**, 536–541.

ABE Y, NAKAMURA M, OHNISHI Y, INABA M, UEYAMA Y AND TAMAOKI N. (1994*b*). Multidrug resistance gene (MDR1) expression in human tumor xenografts. *Int. J. Oncol.*, **5**, 1285–1292.

BAAS F, JONGSMA APM, BROXTERMAN HJ, ARCECI RJ, HOUSMAN D, SCHEFFER GL, RIETHORST A, VAN GROENIGEN M, NIEUWINT AWM AND JOENJE H. (1990). Non-P-glycoprotein expression during *in vitro* selection for doxorubicin resistance in a human lung cancer cell line. *Cancer Res.*, **50**, 5392–5398.

BERGH J, NYREN P AND LARSSON R. (1990). Mechanisms for acquired cytotoxic drug resistance in human small cell lung cancer and the potential utilization of resistance modifiers – a review with focus on *in vitro* studies. *Lung Cancer*, **6**, 9–15.

BRITRAN JD, GOLOMB HM, LITTLE AG AND WEICHSELBAUM RR. (1988). *Lung Cancer, A Comprehensive Treatise*, pp. 173–241 and 307–397. Grune and Stratton: Orland.

CHEN C, CHIN JE, UEDA K, CLARK DP, PASTAN I, GOTTESMAN MM AND ROBINSON IB. (1986). Internal duplication and homology with bacterial transport proteins in the mdr1 (p-glycoprotein) gene from multidrug-resistant human cells. *Cell*, **47**, 381–389.

CHEN C, CLARK DP, UEDA K, PASTAN I, GOTTESMAN MM AND ROBINSON IB. (1990). Genomic organization of the human multidrug resistance (MDR1) gene and origin of P-glycoproteins. *J. Biol. Chem.*, **265**, 506–514.

COLE SPC, DOWNES HF AND SLOVAK ML. (1989). Effect of calcium antagonists on the chemosensitivity of two multidrug-resistant human tumour cell lines which do not overexpress P-glycoprotein. *Br. J. Cancer*, **59**, 42–46.

COLE SPC, BHARDWAJ G, GERLACH JH, MACKIE JE, GRANT CE, ALMQUIST KC, STEWART AJ, KURZ EU, DUNCAN AMV AND DEELEY RG. (1992). Overexpression of a transporter gene in a multidrug-resistant human lung cancer cell line. *Science*, **258**, 1650–1654.

DHINGRA HM, VALDIVIESO M, CARR DT, CHIUTEN DF, FARHA P, MURPHY WK, SPITZER G AND UMSAWASDI T. (1985). Randomized trial of three combinations of cisplatin with vindesine and/or VP-16-213 in the treatment of advanced non-small cell lung cancer. *J. Clin. Oncol.*, **3**, 176–183.

FLENS MJ, IZQUIERDO MA, SCHEFFER GL, FRITZ JM, MEIJER CJLM, SCHEPER RJ AND ZAMAN GJR. (1994). Immunochemical detection of the multidrug resistance-associated protein MRP in human multidrug-resistant tumor cells by monoclonal antibodies. *Cancer Res.*, **54**, 4557–4563.

FOJO AT, WHANG-PENG J, GOTTESMAN MM AND PASTAN I. (1985). Amplification of DNA sequences in human multidrug-resistant KB carcinoma cells. *Proc. Natl Acad. Sci. USA*, **82**, 7661-7665.

GIACCONE G, GAZDAR AF, BECK H, ZUNINO F AND CAPRANICO G. (1992). Multidrug sensitivity phenotype of human lung cancer cells associated with Topoisomerase II expression. *Cancer Res.*, **52**, 1666-1674.

GRANT CE, VALDIMARSSON G, HIPFNER DR, ALMQUIST KC, COLE SPC AND DEELEY RG. (1994). Overexpression of multidrug resistance-associated protein (*MRP*) increases resistance to natural product drugs. *Cancer Res.*, **54**, 357-361.

GROS P, NERIAH YB, CROOP JM AND HOUSMAN DE. (1986). Isolation and expression of a complementary DNA that confers multidrug resistance. *Nature*, **323**, 728-731.

ITOH J, OSAMURA RY AND WATANABE K. (1992). Subcellular visualization of light microscopic specimens by laser scanning microscopy and computer analysis: a new application of image analysis. *J. Histochem. Cytochem.*, **40**, 955-967.

NIEUWINT AWM, BAAS F, WIEGANT J AND JOENJE H. (1992). Cytogenetic alterations associated with P-glycoprotein- and non-P-glycoprotein-mediated multidrug resistance in SW-1573 human lung tumor cell lines. *Cancer Res.*, **52**, 4361-4371.

REEVE JG, RABBITS PH AND TWENTYMAN PR. (1990). Non-P-glycoprotein-mediated multidrug resistance with reduced EGF receptor expression in a human large cell lung cancer cell line. *Br. J. Cancer*, **61**, 851-855.

RICHARDS II F, PERRY DJ, GOUTSOU M, MODEAS C, MUCHMORE E, REGE V, CHAHINIAN AP, HIRSH V, POIESZ B AND GREEN MR. (1991). Chemotherapy with 5-fluorouracil (5-FU) and cisplatin or 5-FU, cisplatin, and vinblastine for advanced non-small cell lung cancer. *Cancer*, **67**, 2974-2979.

RONINSON IB. (1991). *Molecular and Cellular Biology of Multidrug Resistance in Tumor Cells*, pp. 91-104. Plenum: New York.

SAMBROOK J, FRITSH EF AND MANIATIS T. (1989). *Molecular Cloning: A Laboratory Manual*, 2nd edn, p. 7. Cold Spring Harbor Laboratory Press: Cold Spring Harbor, NY.

SLOVAK ML, HOELTGE GA, DALTON WS AND TRENT JM. (1988). Pharmacological and biological evidence for differing mechanisms of doxorubicin resistance in two human tumor cell lines. *Cancer Res.*, **48**, 2793-2797.

THOMAS GA, BARRAND MA, STEWART S, RABBITTS PH, WILLIAMS ED AND TWENTYMAN PR. (1994). Expression of the multidrug resistance-associated protein (*MRP*) gene in human lung tumours and normal tissue as determined by *in situ* hybridisation. *Eur. J. Cancer*, **30A**, 1705-1709.

UEDA K, CLARK DP, CHEN C, ROBINSON IB, GOTTESMAN MM AND PASTAN I. (1987). The human multidrug resistance (mdr1) gene. *J. Biol. Chem.*, **262**, 505-508.

UICC (1978). *TNM Classification of Malignant Tumours*, 3rd edn. International Union Against Cancer: Lyon.

VERSANTVOORT CHM, BROXTERMAN HJ, PINEDO HM, DE VRIES EGE, FELLER N, KUIPER CM AND LANKELMA J. (1992). Energy-dependent processes involved in reduced drug accumulation in multidrug-resistant human lung cancer cell lines without P-glycoprotein expression. *Cancer Res.*, **52**, 17-23.

WHO (1982). The World Health Organization histological typing of lung tumours. *Am. J. Clin. Pathol.*, **77**, 123-136.

WILLIAMS CJ. (1989). Chemotherapy of non-small-cell lung cancer. *Br. J. Cancer*, **60**, 9-11.

ZAMAN GJR, VERSANTVOORT CHM, SMIT JJM, EIJDEMS EWHM, DE HAAS M, SMITH AJ, BROXTERMAN HJ, MULDER NH, DE VRIES EGE, BAAS F AND BORST P. (1993). Analysis of the expression of *MRP*, the gene for a new putative transmembrane drug transporter, in human multidrug resistant lung cancer cell lines. *Cancer Res.*, **53**, 1747-1750.